# Right ventricular apical thrombus formation after transcatheter edge-to-edge mitral valve repair: a case report

**DOI:** 10.3389/fcvm.2025.1640491

**Published:** 2025-12-05

**Authors:** Shuyue Cai, Jun Zhang, Zhongshan Gou, Haifeng Zhang

**Affiliations:** 1 Department of Cardiology, The Affiliated Suzhou Hospital of Nanjing Medical University, Suzhou Municipal Hospital, Gusu School, Suzhou, China; 2 Department of Geriatric Cardiology, The First Affiliated Hospital of Nanjing Medical University, Jiangsu Province Hospital, Nanjing, China

**Keywords:** transcatheter edge-to-edge repair (TEER), right ventricular apical thrombosis, anticoagulant treatment, severe mitral regurgitation, heart failure

## Abstract

Transcatheter edge-to-edge repair is an alternative therapy for patients with severe mitral regurgitation. Here, we report the first case of right ventricular apical thrombus formation following transcatheter edge-to-edge repair in a 54-year-old male with heart failure and reduced ejection fraction. Post-procedural transthoracic echocardiography revealed multiple apical right ventricular thrombi on postoperative day 2. Anticoagulation with warfarin and low-molecular-weight heparin resulted in thrombus resolution, and the patient was discharged uneventfully. This case highlights the importance of vigilant postoperative monitoring and tailored thromboprophylaxis in patients with impaired ventricular function.

## Introduction

TEER has emerged as a significant advancement in MR management, offering a safe and effective alternative to surgical intervention, particularly for high-risk patients (1–3). There have been cases of acute thrombus formation during the mitral valve TEER procedure in the left and right atria: at the site of transseptal puncture, as well as on the clip delivery system/guide catheter and on the mitral valve TEER device itself (4, 5). However, RV thrombosis remains undocumented. This report presents a unique case of RV apical thrombus formation following TEER, emphasizing the importance of continuous monitoring and individualized patient management.

## Case description

A 54-year-old man presented with progressive exertional dyspnea and chest tightness over one year, significantly worsening in the preceding month to NYHA class III limitations. The patient had a 3/4 holosystolic murmur at the left fifth intercostal space at the midclavicular line. He had coarse breath sounds, abnormal carotid pulsation, but no lower extremity edema. Laboratory evaluation demonstrated elevated D-dimer (11.57 mg/L), international normalized ratio (INR) of 2.0, and markedly elevated N-terminal pro-B-type natriuretic peptide (NT-proBNP: 13,000 pg/mL). The patient maintained sinus rhythm throughout the hospitalization. Coronary computed tomography angiography (CCTA) excluded coronary artery disease, and the patient denied prior hypertension, diabetes, or atherosclerotic history.

## Diagnostic assessment and therapeutic intervention

**Figure 1:**
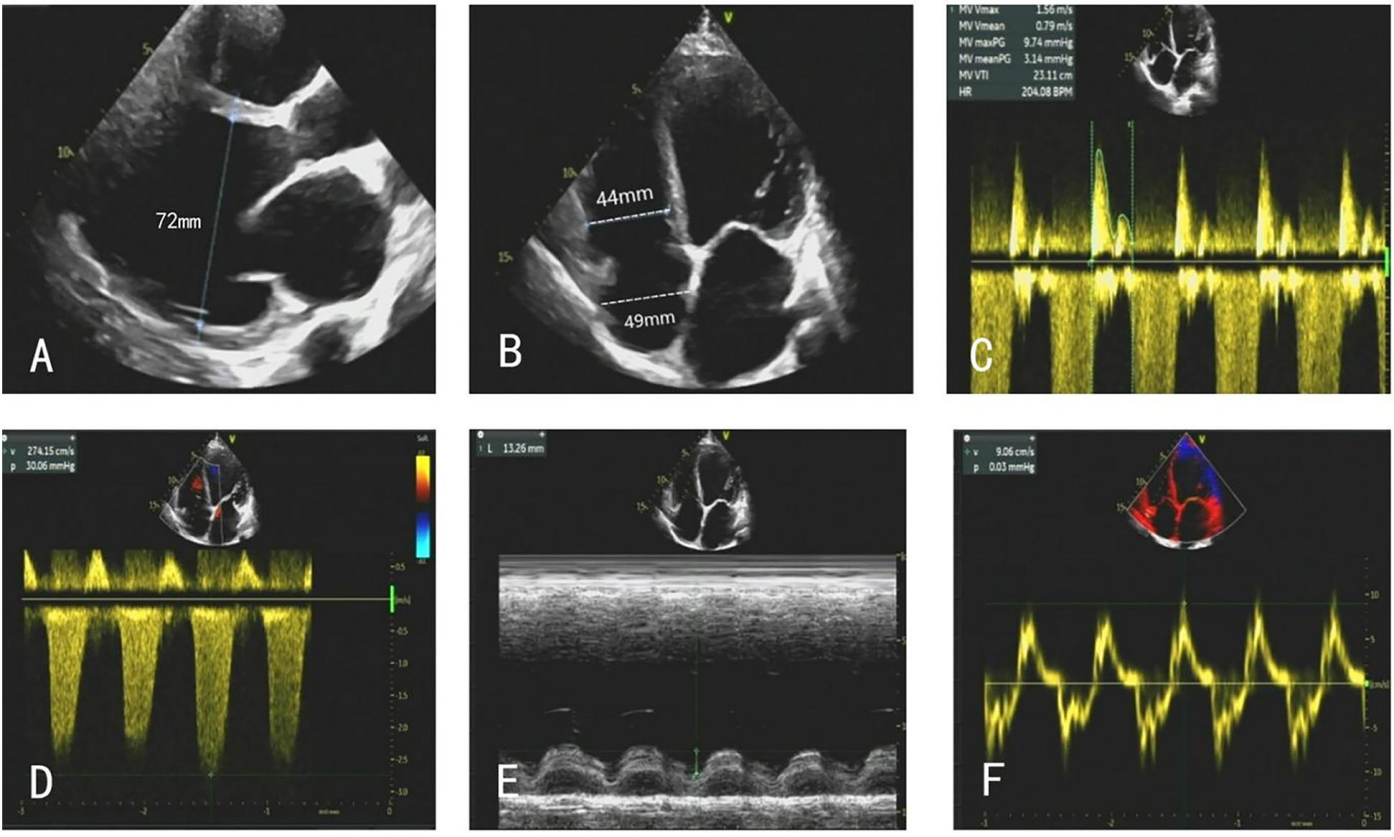
**(A–F)** TTE on admission showed global cardiomegaly, severe MR, and RV dysfunction.

**Figure 2:**
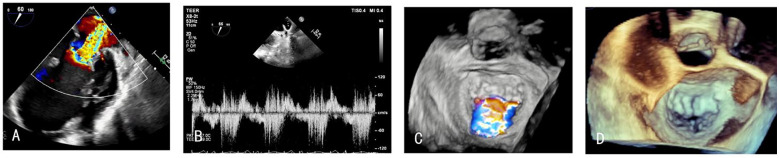
**(A,C,D)** TEE showing the MR between A2 and P2, **(B)** pulmonary vein systolic flow reversal before the procedure.

After admission, the patient underwent a comprehensive cardiac ultrasound assessment, including both transthoracic and transesophageal echocardiography. Preoperative transthoracic echocardiography (TTE) demonstrated global cardiomegaly (LV end-diastolic diameter: 72 mm), severe MR (vena contracta: 12 mm), LV ejection fraction (LVEF) 24%, and RV dysfunction (TAPSE: 13 mm) (Figures 1A–F). The echocardiographic findings, including biventricular dilation and systolic dysfunction in the absence of significant coronary artery disease, were consistent with a diagnosis of dilated cardiomyopathy. Further transesophageal echocardiography (TEE) confirmed the regurgitant jet was wide, between the A2 and P2 scallops, with pulse wave Doppler imaging of the pulmonary vein showing systolic flow reversal (Figure 2).

The patient was treated with guideline-directed medical therapy, including furosemide, sacubitril-valsartan, metoprolol, and spironolactone, but the wheezing symptoms were not significantly improved. After reviewing by a multidisciplinary valve team, the patient was deemed a suitable candidate for mitral valve TEER due to elevated surgical risk, poor functional status, suitable valve morphology, and the presence of severe symptomatic mitral regurgitation. Intraoperative right heart catheterization was performed, revealing a mean pulmonary artery pressure of 38 mmHg, confirming the presence of significant pulmonary hypertension. The procedure proceeded smoothly, intraprocedural echocardiographic guidance used two XTR mitral clips in the A2/P2 position. After release, the regurgitant area was reduced to mild, and the lung vein flow normalized. The average pressure gradient of the MV was 2 mm Hg, and there was no apparent stenosis. Meanwhile The thrombus was not detected intraprocedurally.

Postoperatively, antiplatelet therapy was initiated immediately with aspirin 100 mg and clopidogrel 75 mg daily, along with enoxaparin 4,000 AxaIU subcutaneously twice. On the first postoperative day, repeat coagulation tests showed an INR of 1.68, while the NT-proBNP level decreased to 3,490 pg/mL.

**Figure 3:**
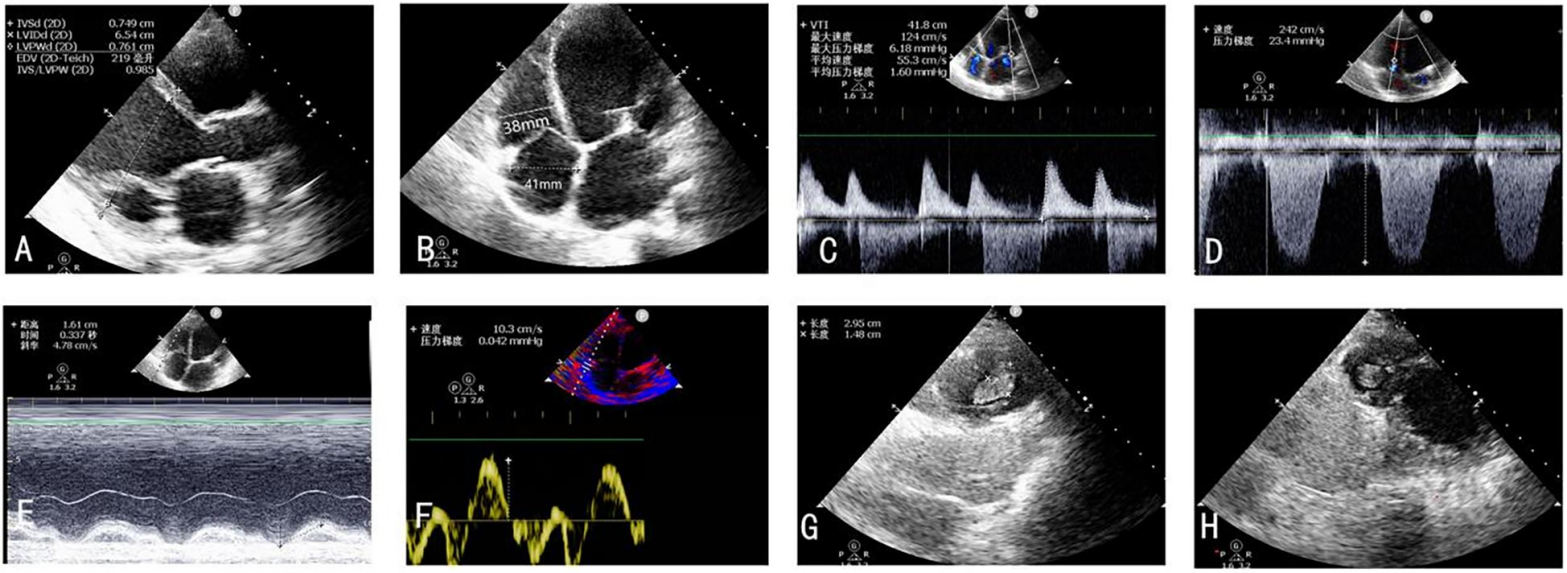
**(A–F)** TTE postoperative day 2 revealed significant improvement in biventricular systolic function alongside favorable hemodynamic changes. **(G,H)** Thrombus forming in the right ventricle on the second postoperative day.

On postoperative day 2, repeat TTE demonstrated significant improvement in biventricular systolic function alongside favorable hemodynamic changes. Mitral regurgitation showed substantial reduction in severity, while tricuspid regurgitation exhibited marked improvement from severe to mild. Pulmonary artery systolic pressure decreased to 25 mm Hg from preoperative measurements of 45 mm Hg. Right ventricular remodeling was evidenced by decreased chamber dimensions following TEER. Right ventricular functional parameters demonstrated modest enhancement: TAPSE increased from 13 mm to 15 mm, while peak systolic tissue Doppler velocity (S′) remained stable at 9 cm/s (Figures 3A–D), and no right-to-left shunt was detected. Notably, echocardiographic evaluation revealed multiple apical thrombi within the right ventricular cavity (Figures 3E–G). Subsequent vascular Doppler studies of the lower extremities and pelvic vasculature demonstrated preserved triphasic flow patterns without evidence of deep venous thrombosis.

**Figure 4:**
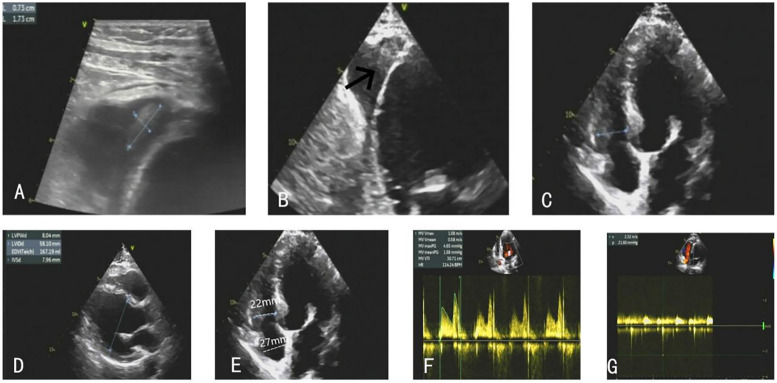
**(A–C)** The right ventricular thrombus gradually eliminated. **(D–G)** TTE on the follow-up showed stable clips with mild MR, and the patient's ventricular systolic function significantly improved.

The patient was promptly initiated on warfarin 2.5 mg daily for anticoagulation and received subcutaneous dalteparin sodium 5,000 IU every 12 h. Serial monitoring of the INR, clinical status, and imaging findings was conducted throughout the follow-up period. The patient remained demonstrating resolution of heart failure symptoms. Follow-up TTE performed during subsequent evaluations revealed progressive reduction in the size of the right ventricular apical thrombi (Figures 4A–C). The patient was discharged on warfarin 2.5 mg daily for long-term thromboprophylaxis, with a target INR range of 2.0–3.0.

At the 1-month follow-up, repeat TTE demonstrated further diminution of the thrombi. After 7 months of anticoagulation therapy, follow-up echocardiography confirmed complete resolution of the thrombus, stable clips with mild MR, and preserved valvular function. At this time, the INR was therapeutic at 2.31, with a mean mitral valve pressure gradient of 1.6 mmHg and a peak mitral inflow velocity of 108 cm/s (Figures 4D–G). No thromboembolic complications or recurrent thrombus formation were observed during the surveillance period, and noparadoxical embolic events occurred, including stroke, systemic infarction, or hypoxemia.

## Discussion

To our knowledge, this is the first case of right ventricular thrombus formation following TEER, an overlooked complication. However, the risk of thrombosis and the best thromboprophylactic therapy remain undetermined at present.

Current evidence suggests a remarkably low incidence of post-TEER ventricular thrombosis, with left ventricular (LV) thrombi reported in 1.1% of cases and no prior RV occurrences (6). To date, thrombogenesis mechanisms remain incompletely understood, proposed mechanisms include synergistic interactions among ventricular dysfunction, hemodynamic alterations, hypercoagulable states, and patient-specific risk factors. Thrombus formation in the LV, particularly in the context of dilated LV, is a known complication in heart failure patients with reduced left ventricular ejection fraction (HFrEF) (7).

The patient had a clearly established predisposition. Pre-operative TTE confirmed severely reduced RV systolic function and dilation, creating a low-flow environment conducive to blood stasis and thrombus formation. This pre-existing RV myopathy is the fundamental underlying risk factor. We propose that the TEER procedure acted as a significant pro-thrombotic stimulus in this vulnerable setting. The acute reduction in left atrial and ventricular filling pressures post-clip deployment alters the loading conditions of the already compromised right heart. This sudden hemodynamic shift could potentially exacerbate relative RV stasis (8). Furthermore, the procedure itself induces a transient inflammatory and hypercoagulable state due to vascular access, device manipulation, and tissue injury, which may have contributed to a systemic pro-thrombotic milieu. Moreover, the significantly elevated D-dimer level raises the possibility of a pre-existing subclinical hypercoagulable state or even a cryptic deep vein thrombosis. Mechanical manipulation during femoral venous access could theoretically have dislodged a small, undetected thrombus, embolizing it to the RV apex where it propagated in the low-flow environment.

Regarding therapeutic alternatives, several theoretical options could be considered for managing right ventricular thrombus, though none are well-established in this specific context. Percutaneous thrombus aspiration or mechanical extraction represents a potential interventional approach, particularly in cases of large, mobile, or high-risk thrombi. However, the procedural risks, including thrombus fragmentation and embolization, must be carefully weighed against the benefits, especially in a patient with recent TEER and impaired ventricular function. Catheter-directed thrombolysis is another option that could be considered in scenarios of hemodynamic compromise or failed anticoagulation, though it carries a significant bleeding risk and lacks evidence in this setting. In our case, given the patient's clinical stability and the absence of embolic events, we opted for a conservative approach with intensified anticoagulation, which ultimately led to complete thrombus resolution. This outcome supports the efficacy of anticoagulation as a first-line strategy in stable patients, while more invasive options may be reserved for those with contraindications to anticoagulation or signs of impending embolism.

Current perioperative antithrombotic strategies for TEER, which typically combine intraprocedural therapeutic anticoagulation with six months of dual antiplatelet therapy post-intervention, remain inadequately supported by clinical evidence (9). LV thrombus formation has been documented despite direct oral anticoagulant (DOAC) therapy, raising questions about their efficacy in this population (6). Emerging data indicate warfarin may offer superior thromboprophylaxis compared to DOACs in TEER patients (6), though recent studies present conflicting conclusions regarding bleeding risks and stroke prevention (10–12). Consequently, optimal thromboprophylaxis strategies remain undefined, necessitating individualized risk-benefit assessments.

## Limitations

No preoperative Doppler ultrasound was performed to systematically exclude deep vein thrombosis; however, no obvious DVT was noted during femoral venous access. Preoperative cardiac CT was not performed, but comprehensive TEE before, during, and after the procedure showed no right ventricular thrombus. An SGLT2 inhibitor was not prescribed during initial GDMT optimization, representing a limitation in medical therapy. While the elevated D-dimer was primarily attributed to heart failure, a comprehensive venous duplex ultrasound at admission would have been more complete.

## Patient perspective

From a patient's point of view, the described solution was accepted. More than one year after the procedure, the patient has no complaints, and he relieved heart failure symptoms.

## Data Availability

The original contributions presented in the study are included in the article/Supplementary Material, further inquiries can be directed to the corresponding author.
