# Supplementary material for: Right ventricular apical thrombus formation after transcatheter edge-to-edge mitral valve repair: a case report
Source: Front Cardiovasc Med. 2025 Dec 5;12:1640491. doi: 10.3389/fcvm.2025.1640491 (PMC12714993; doi:10.3389/fcvm.2025.1640491)
Supplement: Supplementary file 1 [file Table1.docx]

**Timeline**

| ****Time Point**** | ****Clinical Event**** | ****Key Findings/Interventions**** |
| --- | --- | --- |
| ****~1 year prior**** | Symptom onset | Progressive exertional dyspnea and chest tightness |
| ****~1 month prior**** | Symptom worsening | Dyspnea worsened to NYHA class III |
| ****Admission (Day 0)**** | Initial presentation | Holosystolic murmur (3/4), coarse breath sounds, abnormal carotid pulsation |
|  | Transesophageal echocardiography (TEE) | Severe MR between A2/P2 scallops, systolic flow reversal in pulmonary vein |
| ****Pre-TEER**** | Guideline-directed medical therapy | Furosemide, sacubitril-valsartan, metoprolol, spironolactone |
| ****TEER Procedure**** | Mitral valve repair | 2 XTR clips implanted in A2/P2 position |
| ****Post-op Day 1**** | Antiplatelet therapy initiation | Aspirin 100mg + Clopidogrel 75mg daily + Enoxaparin 4000 AxaIU BID |
| ****Post-op Day 2**** | Routine TTE | ****Unexpected finding:**** Multiple apical RV thrombi (Fig 3E-G) ****Improvements:**** ↑Biventricular function, TR severe→mild, PASP 25mmHg (↓from 45mmHg), TAPSE ↑to 15mm |
| ****Post-op Day 2**** (action) | Anticoagulation initiation | ****Warfarin 2.5mg daily**** + ****Dalteparin sodium 5000 IU SC q12h**** (target INR 2.0-3.0) |
| ****Hospital stay**** | Monitoring | Serial INR, clinical status, and TTE |
| ****Discharge**** | Discharged on anticoagulation | Warfarin 2.5mg daily |
| ****1-month follow-up**** | Outpatient TTE | Further reduction in RV thrombus size |
| ****7-month follow-up**** | Final assessment | ****Complete thrombus resolution**** Stable clips, mild MR, preserved valve function |
